# Create Solidarity Networks: Dialogs in Reddit to Overcome Depression and Suicidal Ideation among Males

**DOI:** 10.3390/ijerph182211927

**Published:** 2021-11-13

**Authors:** Gisela Redondo-Sama, Teresa Morlà-Folch, Ana Burgués, Jelen Amador, Sveva Magaraggia

**Affiliations:** 1 Department of Pedagogy, University Rovira i Virgili, 43007 Tarragona, Spain; 2 Department of Business Management, University Rovira i Virgili, 43204 Reus, Spain; teresa.morla@urv.cat; 3 Department of Sociology, University of Granada, 18001 Granada, Spain; anaburgues@ugr.es; 4 Department of Sociology, Autonomous University of Barcelona, 08193 Bellaterra, Spain; Mariajerusalen.Amador@uab.cat; 5 Department of Sociology and Social Research, University of Milan-Bicocca, 20126 Milan, Italy; sveva.magaraggia@unimib.it

**Keywords:** masculinity, suicide, suicide ideation, communicative content analysis, social media analytics, social media

## Abstract

The emerging scientific literature examines masculinity and gender roles as risk factors for suicide ideation or suicide in young adults and adolescents. In this vein, recent studies show that certain traditional masculine norms are related to poorer mental health-related outcomes, which influences suicide and suicide ideation. This study contributes with new understandings about the associations between masculinity and suicidal ideation among males through Reddit debates in English. The posts with more interactions referring to masculinity in the topics gender and education have been selected on Reddit, emphasizing transformative personal experiences potentially helping avoid suicide ideation. Through the analysis of Reddit posts, it is shown how users can generate spaces to express the diverse ways to live with masculinity. The discussions on Reddit in the different areas selected demonstrate the existence of proposals on how to overcome fears and facilitate relaxation of norms regarding self-reliance to encourage help-seeking when feeling depressed and therefore at greater risk of suicide ideation. The results highlight the potential importance of platforms such as Reddit to create solidarity networks, showing multiple ways of being a man and demystifying dominant masculinity by sharing different experiences.

## 1. Introduction

Suicide is a serious public health problem. Every year, around 703,000 people worldwide take their own lives, and a large number of people, especially teenagers, report having suicidal ideation [1]. In fact, suicide is one of the leading causes of death in young people. In the United States, suicide was the second leading cause of death in 2019 among individuals between the ages of 10 and 34 [2], and in Australia, suicide was the first cause of death in people between 15 and 44 years in the period from 2017 to 2019 [3]. In 2019, suicide was the fourth leading cause of death among people between 15 and 29 years worldwide [1]. It is important to highlight that this is a gendered phenomenon; men have a higher suicide rate than women. For example, in 2019 in the United States, male suicides occurred 3.63 times more often than among women [4]. Behind the data about suicide, a major concern is to identify and prevent suicide ideation.

Suicidal ideation is an important risk factor for suicide, but this is poorly understood among adolescent males [5,6]. Suicide ideation and attempting suicide affect the emotional lives of millions of men, damaging their well-being and their families [7]. In light of this unresolved global health problem, the suicide mortality rate is an indicator of Target 3.4 of the Sustainable Development Goals for 2030 [8], aimed at ensuring healthy lives and promoting well-being for everyone at all ages.

The emerging scientific literature shows that certain traditional masculine roles are linked to poorer mental health-related outcomes. Rigid cultural norms that imply heteronormativity and homophobia have negative consequences for mental and physical health [9]. Suicidality is a critical health issue among Sexual and Gender Minority (SGM) youths, such as young gay, lesbian, bisexual, transgender and questioning (LGBTQ) individuals [9,10]. Moreover, suicidal thoughts are the most common manifestation of distress in the form of speculation of wanting to end one’s life after years of cumulative abuses of transphobic violence [11,12]. It is also relevant to take into account that research consistently indicates increased suicide risk in indigenous and military men, including veterans in the middle years, who have a higher suicide rate than their peers who have not served in the military [7,13,14,15]. A study on young men aged 18–30 who committed suicide in Norway theorized that death by suicide represented an “act of masculinity” linked to the perceived failure to respond to idealized masculine standards [16]. Masculine standards are culturally specific, but usually they are aligned with the traditional models of masculinity [17,18]. These are models that are important to overcome by generating other models, currently conceptualized in the scientific literature as New Alternative Masculinities (NAM) [19,20]. The scientific literature presents the differentiation between the Dominant Traditional Masculinity (DTM) and the Oppressive Traditional Masculinity (OTM). DTM is linked to an image of aggressive and chauvinist manhood. In contrast, OTM represents “nice boys”, who are too nice, are the stereotypical “man to marry” and are for those who want submissive boys. However, this dichotomy is overcome by NAM, where the feelings of passion and respect come together in the same person beyond double standards. The existence of alternatives to traditional masculinities (DTM and OTM) have emerged in the diverse social dimensions of daily life, including virtual interactions and social networks.

Nowadays, social networks contribute to interconnecting people through the Internet in a simple and fast way, facilitating the initiation and growth of social relations. Likewise, it is widely recognized that social networks and loneliness have effects on health. Rico-Uribe et al. [21] identified a stronger relationship between loneliness and health in the younger population than in older people. The previous literature has analyzed how to advance toward the detection of depression on social media, as this may become the first step against suicidal behavior [22,23].

In this arena, this study focuses on the content analysis of messages with a focus on solidarity between Reddit members. The analysis of debates on Reddit provides evidence to advance knowledge about the associations between masculinity and suicidal ideation among males. The results shed light on the existence of a type of alternative behavior that fosters better health habits in men and can generate an online network of support.

### 1.1. Early Detection of Suicide, Suicide Ideation and Support

Early detection and intervention in mental health are crucial in influencing trajectories and preventing life course recurrence [24,25]. However, the prevention of suicide has not been adequately addressed due to a loss of awareness of suicide as a crucial public health problem and the taboo in many communities for having open discussions about it [1,26].

King et al. [5] pointed out that maximizing adolescent health is key to optimizing adult health and well-being. However, the literature shows health service disengagement by men. For instance, Schlichthorst et al. [27] showed that 61% of Australian men do not access regular health check-up visits, signaling a major lost opportunity for preventative mental health discussions. Similarly, studies in different parts of the world emphasize this idea. Men’s resistance to promoting their health and unwillingness to promote professional services followed the constrained choices that flowed from aligning to masculine ideals of self-reliance, competitiveness and aggression [28]. In the same arena, studies estimate that only 25% of sexuality minority men who attempt suicide have accessed professional help and that many are reluctant to seek help from friends, family or other nonprofessionals. Men can perceive seeking help as conflicting with the traditional male qualities of strength, independence and emotional control [4,29,30]. Thus, the literature suggests a critical missed opportunity for early identification and intervention.

Oliffe and Han [31] analyzed interconnected issues to advance toward the well-being of men, including the understanding of connections between masculinities and men’s work-related depression and suicide. Spencer-Thomas [32] recommended using messages that emphasize self-reliance and self-help rather than help seeking, as well as teaching intimate partners, coworkers and others how to recognize and respond to suicide risk in men. In the face of this reality, to tackle male mental health, proactive models of engagement are crucial to preventing suicide. In this line, the study of Wang, Ko, Hsiao, Chen, Lin and Yen [14] (p. 10) revealed “that low family support, but not peer support, during childhood increased the risk of suicidality in emerging adulthood among gay and bisexual men”. Therefore, the role of the family, peers, school staff and friends is crucial to promote men’s well-being and prevent suicide [33,34].

According to the scientific literature, it is relevant to pay attention to educational environments [35]. Therefore, it is essential to examine thoughtful physical well-being at the institutional level, such as by generating an inclusive climate in schools and colleges and having suicide prevention programs [9]. Generating more friendly environments in educational centers and possibilities to, for example, successfully tackle transphobic violence [36], can be useful. In short, suicide prevention is most likely to be effective if a mixture of evidenced-based strategies is used at both the individual and community levels [37].

### 1.2. Solidarity Networks and Accessing Help Online

The advent of Internet and social networks has undoubtedly facilitated the broad circulation and dissemination of information, opening up more opportunities for users to access, interact and produce content. The power no longer lies in having access to information but in managing it, as the information society is characterized by an overabundance of data. Nowadays, citizens can access the most recent scientific evidence on social media and, indeed, channels of communication between science and social or personal networks have improved [38,39]. The ability to connect immediately with friends, relatives and strangers alike has transformed the way social relationships are created and maintained and, as a consequence, altered the structure of our social fabric [40]. Some authors highlighted that the interconnection between people makes their health interconnected [41], while others showed how social networking and online social networking benefit patients [42,43,44]. In fact, recent studies showed that young men may have a preference for accessing online help [45,46]. In a study conducted in the UK, Australia and New Zealand, the authors showed the following: “In 10% of qualitative studies, young people identified opportunities to communicate distress and attend treatment via digital tools as facilitators to seeking/accessing treatment” [47] (p. 206). Moreover, Kim et al. [48] demonstrated the role of social networks in maximizing population behavior change.

Online communication channels are becoming a new way for people to express their suicidal tendencies [23]. Resonating with the recent works by Losada and Crestani [49] and Leiva and Freire [22], we focus on the social network Reddit.

## 2. Materials and Methods

This article is part of the fieldwork carried out as a section of the project “ALLINTERACT, Widening and diversifying citizen engagement in science” (European Commission, H2020, SwafS-20-2018-2019). From October 2020 to May 2023, the project aims, on the one hand, to create new knowledge about how to transform potential citizen participation in science into actual engagement in scientific research, and on the other hand, to unveil new ways to engage societal actors, including young citizens and groups that have traditionally been excluded from science (e.g., youth). The ALLINTERACT project focuses on two Sustainable Development Goals: quality education and gender equality. On the basis of these two areas (gender and education), this article focuses on the results related to the associations between masculinity and suicidal ideation among males.

This contribution focuses on the Social Media Analytics (SMA) of the social network Reddit to analyze new understandings of the associations between masculinity and suicidal ideation among males through debates on Reddit. Reddit is a registered online community that aggregates social news and online discussions. Reddit has no limitation regarding the number of characters. It consists of many topic categories, and each area of interest within a topic is called a subreddit. For example, one subreddit is “SuicideWatch: peer support for anyone struggling with suicidal thoughts”. When developing a search on Reddit, it is possible to obtain results through posts and communities or users. As Reddit does not use hashtags, the results show all posts that use the keyword in every public reddit. The discussions used were in English.

The study is based on the Communicative Content Analysis [50], following the postulations of the communicative methodology [51]. This methodology is based on a dialogic co-creation of knowledge between researchers and citizens. The communicative methodology has been demonstrated to achieve social impacts in different fields of knowledge since it was conceptualized, contributing to tackling social challenges [52].

For the extraction and use of Reddit posts, we have followed the platform’s rules, regulated in the Reddit User Agreement [53]. In accordance with this, no users are identified, nor are any posts quoted literally to guarantee the anonymity of the texts. We used the Reddit API and Python to obtain the message data.

### 2.1. Research Design

The aim of the research design was to analyze the ways in which discourses on suicide and mental health were generally approached in Reddit conversations. Therefore, instead of narrowing the search to identify conversations about mental health and suicide, the study focused on how discourses linked to the research topics emerged in conversations on gender and education. We used the *top-down* strategy to identify the relevance of the research goals through collecting citizens’ voices on social media [54]. This strategy consisted of the definition of keywords related to the field of study, in this case gender and education. Then, the next step was to contrast if the keywords were expressed by citizens in their opinions, comments and posts expressed on social media. To this aim, we identified the communities in which users shared posts and commented on posts about different topics. Five criteria underlined the selection of subreddits to be analyzed:

- Interaction through debate;
- Representation of diverse citizens in terms of age, gender, educational background or cultural background, among other factors;
- Outreach of the community (number of members);
- High level of interaction with users (including likes, posts and comments);
- Presence of filter categories that included topics related to this research (e.g., health, anxiety, depression and suicide).

The final selected pages were obtained on 15 March 2021, including 5 subreddits for gender and 5 subreddits for education (see Table 1). Each subreddit deals with a specific topic. People debate about it, and the debates are not directly related to masculinity or mental health. However, the topics emerge and are discussed in the framework of other discussions. Table 1 includes the topics of each subreddit selected.

### 2.2. Data Analysis

The analysis was based on the following four steps:

1. The researchers anonymized all the data.
2. The researchers checked manually that the selected messages met the inclusion criteria and discarded the others, discarding duplicated messages and messages posted by fake accounts or bots.
3. The researchers compiled all messages that met the inclusion criteria in an Excel file.
4. The researchers agreed upon the keywords to select the posts. In this study, the keywords were anxiety, depression, health, suicide and a combination of them. The elaboration of the codebook was dialogic, combining predefined categories with those categories that emerged during the analysis. All the posts containing one (or more) of these keywords were compiled into a single Excel file for further coding. Table 2 summarizes the number of posts identified for each keyword in each subreddit.

The analysis included 958 posts written by English-speaking communities. The Reddit discussions selected were not directly related to the topic of mental health or suicide, as they were extracted from regular conversations about gender and education. Therefore, 470 posts were selected, discarding 488 posts and Reddits for not being directly related to the topic under study. The keyword that was discarded the most was “health”, as these posts were related to questions about healthy living and concerns about health in the COVID-19 context. Table 2 summarizes the subreddits and keywords selected for the analysis.

The authors of this article guaranteed accurate analysis of the data collected in dialogue with the research team. Once the 958 messages were coded, the results obtained were discussed. It should be noted that the selected and coded 470 total posts were written by both women and men, and according to the aim of this study, in the results, we include comments we identified that were written by men. In order to develop the Communicative Content Analysis, the posts were codified by categories without first differentiating between men and women, as in many cases, the person who wrote the post did not specify the gender, and the English language did not allow for identification. Then, an in-depth dialogic data analysis of each post was developed, identifying qualitatively those discourses in posts clearly expressed by men. According to Reddit rules and anonymity, the messages cannot be displayed, and therefore, they are explained but not transcribed in the results.

In the first round of analysis, the 470 selected posts were categorized according to the type of shared experience. In this sense, we found seven categories of posts:

1. Posts that did not comment on personal experiences but spoke generally;
2. Those that directly asked for advice;
3. Those who commented on their own experiences;
4. Those posts that were responding through their own experiences;
5. Those who commented on someone else’s experience;
6. Those who responded and their arguments were based on someone else’s experience;
7. Those who responded without commenting on any experience.

**Figure 1:**
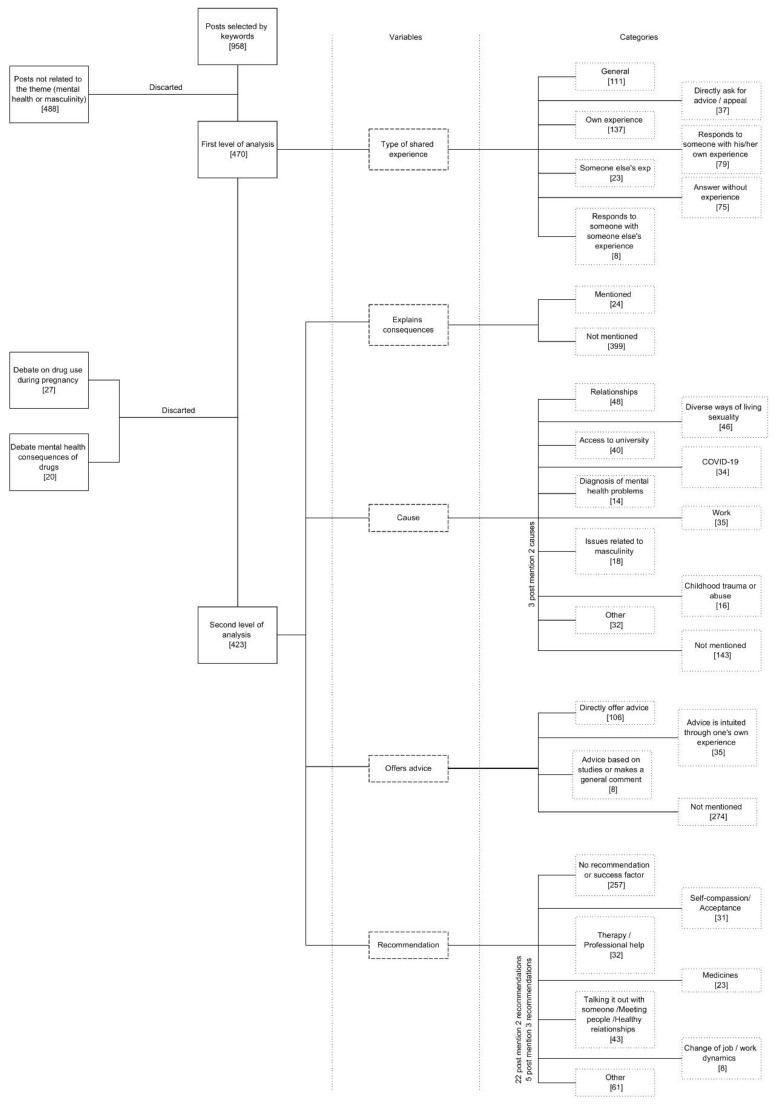
Number of posts by variables and categories.

After this first round of analysis, we separated and discarded those posts that dealt with the debate on drugs during pregnancy (27 posts) and those that commented on the consequences of drugs on mental health (20 posts). The first round was used to determine the four variables for categorizing the posts in the second round (Figure 1). Therefore, in the second round, 423 posts were classified with the following variables (Table 3): (1) explain consequences, (2) causes, (3) offer advice and (4) recommendations. Each variable had different categories which contained the corresponding numbers of the coding scheme:

Variable 1 Explained the consequences of their mental health problems with two categories of analysis: mentions (1.1) or does not mention consequences (1.2).
Variable 2 Causes that they identified with the origin or aggravation of their mental health problems. The analysis categories that finally resulted were relationships (2.1), diverse ways of living sexuality (2.2), access to university (2.3), COVID-19 (2.4), diagnoses without apparent cause (2.5), work (2.6), aspects related to their masculinity (2.7), childhood trauma or abuse (2.8), other causes (2.9) and not mentioned (2.10).
Variable 3 Whether and what kind of advice was offered. The four categories were directly offered advice (3.1), advice can be intuited through one’s own experience (3.2), advice offered is based on studies or is a general comment (3.3) and not mentioned (3.4).
Variable 4 Whether and what kind of recommendations were made. The seven categories were if they did not mention a recommendation and if they did (4.1), self-compassion or acceptance (4.2), therapy or asking for professional help (4.3), medicines and drugs (4.4), improving their social relations or discussing their problems with someone (4.5), change of job or work dynamics (4.6) and the last category grouping other types of recommendations (4.7).

## 3. Results

The analysis of 470 posts of the first round related to mental health showed that in 216 cases, the posts included the personal experiences of the persons who wrote the posts, either explaining their situations (137 posts) or responding to other posts (79 posts). It is worth noting that 45 out of 470 posts emphasized suicide, even though they were not in subreddits dedicated to the topic.

In the second round, the qualitative analysis of 423 posts resulted in the selection of 49 posts to illustrate the results, which are also explained in this section in relation to the first round. These 49 posts were classified according to the following coding scheme (Table 4).

In the analysis, the posts with transformative comments were highlighted, as they were crucial to help overcome situations of potential suicide and suicide ideation. These comments could contribute to overcoming strong negative feelings of anxiety and hopelessness that may lead to suicide or its ideation [23]. In this line of thought, the analysis of the posts showed that participants talked about toxic masculinity and how this was probably a cause of suicide (e.g., Posts 43, 45–47). The posts also highlighted how the patriarchy especially oppresses those men who do not fit into the masculine value narrative (Post 49). More than 46% of the users exposed their experiences. Furthermore, some of the comments in the posts were in line with the data of the American Foundation for Suicide Prevention [4], which points out that male suicide rates are three to four times higher than female suicide rates (Post 47).

The results are detailed below in two sections. First, we highlight the results that demonstrate the solidarity generated on Reddit around the concept of mental health. Second, we emphasize those mental health interactions that challenge masculinity by showing the oppression that some of these dominant or oppressive models generate for men.

### 3.1. Reddit as a Support Network

The analysis of posts demonstrates that Reddit is a network for sharing experiences. In 137 posts, participants commented on their own experiences and personal issues related directly or indirectly to their mental health. Usually, the posts and comments focused on the causes of their problems; only 24 posts mentioned the consequences of their states of mind, and 280 mentioned the causes. The analysis noticed that mentioning the causes made it easier for the community to offer advice on how to overcome situations. Twenty-four people commented directly on the consequences of mental health problems and how they affected their lives. For example, Post 184 detailed feeling severely depressed and empty, and others commented on other consequences such as not looking straight into the eyes (Post 138), back pain (Post 244), difficulty sleeping (Post 268) or difficulty memorizing (Posts 259 and 301).

The platform became a space for support among participants. In the posts analyzed, 37 were mainly aimed at asking for advice directly, appealing to other users’ experiences and the need to know similar experiences to encourage each other and overcome the feeling of incomprehension. In general, the response of the community was excellent, since 106 posts offered direct recommendations or advice. The vast majority of the responses aimed to share experiences that could help others to cope with the situation and feeling of incomprehension. Furthermore, the responses encouraged users to face their situations and accept themselves as they were (31 posts). Mainly, they were advised to meet new people or improve existing relationships, with the idea of having interactions that benefitted them (43 posts). Participants were also advised to visit specialists to go deep into the problem or overcome anxiety (32 posts).

In the deep analysis of 423 posts, solidarity emerged and prevailed, and each participant exposed his experience or opinion complementing the previous posts. Thus, most of the analyzed posts were characterized by a climate of respect and support among members. The posts that were ironic, critical or offered advice that could be harmful were classified as “exclusionary”. This type of post only represented 4% of the posts (with only 17 posts out of 423). Most of the comments were grateful, with people responding with their experiences as all the ones they had read had been “helpful” (e.g., Posts 274 and 605). Another example is Post 15, which stated “I will” after the interaction with the network. Posts 66 and 910 recognized that chatting on Reddit could make people feel better, improving their moods. In other words, Reddit became a space for dialogue and empowerment to deal with problems.

Through the shared experiences, participants sought support and encouraged other users to write similar testimonies (Post 917). Some participants commented that writing in the post made them feel better. Others recognized that sharing their experiences helped them because they did not have other people to share their concerns with. For example, in the headings of Posts 182 and 245, comments stated that the comments were a personal reflection, but that they wanted to share them as the community was friendly. On some occasions, the comments were even thankful (e.g., Posts 12, 169, 223, 293, 545, 605, 897 and 953). Post 271 was edited, adding that the comments were helping him to improve his anxiety. The analysis also showed that sharing posts contributed to the feeling of being part of a community, or at least that someone understood them. Post 537, for example, thanked the community for their ideas and encouragement. The edited post commented that he felt “more validated” among the Reddit community than in his school. Others appreciated that they dared to share their experiences with the community (e.g., Post 277). When people talked about their lives, they explained the precise details of their experiences. They may have explained things on the subreddit networks that they would not explain in person, not even to their inner circles. They explained issues from their past and present situations and their actions and thoughts, such as the relationships they had or the expectations society had of them as men. The different conversations generated spaces of free expression where people could talk about their sexuality, opening a forum where men could express their ways of living with their masculinity and find support in the community.

### 3.2. Dialogs to Overcome Dominant and Oppressive Traditional Masculinities

Focusing on the factors that people related to a worsening mental situation, most of the posts identified a single reason (277 posts), and only 3 posts exposed multiple factors. It is relevant to highlight that the users directly related the masculinity model as a cause of mental health problems in 18 posts. However, the Communicative Content Analysis revealed that the concerns about masculinity models appeared indirectly in posts under other categories, expanding its presence in Reddit debates and conversations about questioning sexual identity, couple relationships, stress caused by accessing the university or the consequences of the pandemic, among other topics.

The analysis showed that the first cause for worsening mental health was relationships (48 posts). In this vein, 29 participants said that the cause for worsening their mental state was unhealthy affective and sexual relationships. According to this, Post 172 stood out, in which a man commented that his partner made him *feel unhappy and, although he still loved her, he left the relationship* because if he did not make this decision, the situation would lead to suicide. The second cause of a negative mental health impact was the various ways of experiencing sexuality (46 posts). Concerning this, most of the posts referred to their doubts about homosexuality and bisexuality. Several young boys presented their doubts about masculinity. In different posts, the boys mentioned that they did not fit the characteristics of traditional masculinity. Through their stories, it is clear how this created incomprehension and anxiety.

There were posts that referred to questioning the sexual orientation of boys (e.g., Posts 179 and 182). For example, a young man expressed doubts about his sexuality. In his story, he stated that he had a girlfriend and was really in love, but he said that his brother-in-law always described him as gay and stated that anxiety was taking him over because this raised doubts about his sexual orientation. However, the same user defended that everyone should love whoever they want and not be questioned for it (Post 182). This final statement was possibly in a supportive context, where other users encouraged him to face the situation described.

Concerning relationships, men exposed their anxiety about not fitting the stereotypes. The stories in the posts showed the two traditional masculinities: Dominant Traditional Masculinities (DTM) on the one hand and Oppressive Traditional Masculinities (OTM) on the other. Along this line, the posts exposed the reality around traditional masculinity by presenting men who were super romantic and loving creatures that loved women. Still, at the same time, they were men considered as fools (Post 506), which could be qualified as OTM. There were very few cases (Post 168) where sporadic or utilitarian relationships were promoted. However, also overcoming the dichotomy of bad man–passionate (DTM) and good man—boring (OTM), the Reddit posts showed that New Alternative Masculinities (NAMs) emerged. For example, there were two posts where the participants explained the experience of being married to their wives for 10 years but were still entirely in love, highlighting the importance of dialogue to overcome concerns and insecurities and have an honest and passionate relationship at the same time (Posts 939 and 956). Along this line, regardless of their sexual orientation, the comments that spoke of romantic and sexual attraction (e.g., post 179) stood out.

Different posts on Reddit underlined the need to advance to make visible new models of masculinity (Post 459) to promote alternatives to certain dominant and oppressive traditionally masculine roles, make them attractive and not reproduce dominant or oppressive traditional masculinity. Men emphasized that not all men were the same. They specified that not all men made women suffer but claimed to be men in love with their partners, with passionate relationships that rejected violence. However, the users detailed that it was not enough to have healthy levels of confidence and self-esteem (Posts 370 and 411). Thus, there were several reflections on the pressure for men to be the best at everything (Post 411). Therefore, the posts also highlighted the relevance of creating new models of masculinity from a young age to avoid frustrations. For example, in one post based on the user’s own experience with his family, a man stated that he was often told that when a child cried, he had to “act like a man” (Post 48). In this sense, and in relation to socialization processes, some posts highlighted how it is not well seen that men show their feelings (Post 48 and 153). However, Post 48 did not stop explaining this situation and instead described how to reverse it. He paid special attention to the environment and highlighted the relevance to acting in cases like this in his account. The comment argued that the problem lied in the fact that the child’s background did not intervene. The user encouraged creating male solidarity and intervening to respond to comments like this to prevent them from remaining in the daily story (Post 48).

To sum up, Reddit users elaborated on the mental health effects of the dominant and oppressive narrative regarding traditional masculinities. For example, a record is very present in the media (Post 49). It stands out for being an alternative space where participants develop their concerns and show other masculine characteristics that go beyond the stereotypes of traditional masculinity.

## 4. Conclusions

Ensuring healthy lives and promoting well-being at all ages is essential for the Sustainable Development Goal linked to health [8]. Therefore, it is necessary to address a worldwide public health problem such as high suicide rates by understanding the underlying causes. Reddit participants show concern about this issue, as there is a subreddit specifically for peer support for anyone struggling with suicidal thoughts: SuicideWatch, which has more than 289,500 members. Moreover, the topic of suicide and related concerns is also widely addressed in other subreddits, such as in the case of the 10 subreddits analyzed in this article: (1) ApplyingToCollege, (2) education, (3) teaching, (4) science, (5) Teachers, (6) Feminisms, (7) FeMRADebates, (8) bisexual_, (9) Feminism and (10) PurplePillDebate. Among the 10 analyzed subreddits of general topics related to education and gender, we identified 45 posts directly addressing suicide. However, 161 posts related to mental health talked about anxiety, which is highly remarkable since, as the previous literature showed, suicidal individuals express strong negative feelings, anxiety and hopelessness [23].

The findings from the current study show that relationships and diverse ways of living in terms of sexuality are two key dimensions of men’s anxiety and mental health. The findings obtained are aligned with previous studies ([9,10]) demonstrating that heternormativity and homophobia have negative consequences for mental health, and participants on Reddit developed this in their comments. Therefore, in this context, it is relevant to incorporate new models of masculinity to improve men’s health status to overcome insecurities about masculinity [20]. Our results advance the knowledge from the previous literature [5], highlighting the potential relevance of presenting multiple ways of being a male among adolescents. The different posts on Reddit facilitated relaxation of roles regarding self-reliance and dismantling heteronormative masculine roles, offering men who felt passionate in their relationships. Reddit, from these experiences, exposes different ways of living with masculinity. At the same time, it shows how Reddit can be a dialogic space that gives rise to overcoming and transforming stories, which are specified experiences that can help overcome stereotypes. Above all, Reddit becomes a space where there is respect for expressing oneself. As seen in the results, the exclusionary posts are very limited, and the solidarity posts stand out. A remarkable aspect is the existence of advice for men to overcome insecurities and doubts about their masculinity. According to the existing literature, this is a crucial aspect, since men participate less in mental health services, thus missing out on services for suicide prevention and overcoming mental illness [55].

As detailed by the World Health Organization [1], the approach to suicide prevention has to go beyond the health sector and requires an innovative and comprehensive multisectoral approach, working together with the health sectors and other domains, such as social networks. In this arena, Reddit can be another space to detect and help to prevent and overcome suicidal behaviors. The results highlight the potential importance of platforms such as Reddit in creating solidarity networks, showing multiple ways of being a man and demystifying dominant masculinity from sharing different experiences. However, suicide prevention remains an important challenge worldwide. Therefore, it is essential to develop new methods to prevent suicidal ideation and continue exploring the possibilities that social networks such as Reddit can generate to join efforts in suicide prevention.

The major limitation of this study was the use of “gender” and “education” as the main areas underlying the search of Reddit. Although the discussions and debates about masculinities appeared in posts linked to these main two areas, further research is needed to specifically explore the term “masculinity” in Reddit. The number of posts (18) and the qualitative analysis indicate the emerging relevance of the research topic and the need to continue investigating the use of social media to better understand their role in preventing suicidal ideation and overcoming depression among males. In this arena, another limitation of the study refers to the privacy and availability of data for the user profiles in Reddit. The development of strategies to further understand the profiles of users and Reddit dynamics and debates will serve to deeply approach how social networks play a relevant role to improve mental health linked to masculinities.

## Figures and Tables

**Table 1:** Subreddits selected.

Subreddit	About the Community	Total Posts (Including Comments) Analyzed
**Education**		
ApplyingToCollege	Includes questions, advice and discussions on college admissions, such as college essays, scholarships, SAT and ACT test preparation or career guidance.	9255
Education	Brings broad debates on policy, research and technology in educational contexts.	8710
Teaching	News, resources, concerns and advice for teachers at all levels of education are shared.	1069
Science	A subreddit to share and discuss new scientific research from all disciplines.	10,000
Teachers	Wide network on teachers’ concerns, with general questions raised for discussion among users.	9061
**Gender**		
Feminisms	Defined as a safe space for respectful and collaborative debate about feminism, including intersectionality. It details that the content is actively curated by a pro-feminist moderation team.	66
FeMRADebates	Discusses feminism, egalitarianism and men’s rights activism.	260
Bisexual	Defines itself as a subreddit addressed to all those people who are not heterosexual or anyone who does not quite fit into the binary pattern of “straight” and “gay”.	8710
Feminism	In general, in this group, personal issues are addressed from a gender and feminist perspective.	983
PurplePillDebate	Defines itself as a neutral community to discuss sex and gender issues. Specifically, questions are raised in relation to sex and attractiveness.	10,000

**Table 2:** Posts analyzed by keyword and subreddit.

Keywords	Applying ToCollege	Bisexual	Education	Feminism	Feminisms	FeMRADebates	PurplePillDebate	Science	Teachers	Teaching	Total	Not Applicable	Total Analyzed
anxiety	26	34	8	0	0	0	13	59	26	15	181	20	161
depression	11	9	0	0	0	0	13	18	10	4	65	15	50
health	64	50	9	17	0	6	166	77	213	13	615	446	169
suicide	2	1	2	7	0	1	16	4	3	13	49	4	45
combination	7	7	1	0	0	0	7	11	12	3	48	3	45
Total	110	101	20	24	0	7	215	169	264	48	958	488	470

**Table 3:** Coding scheme and number of posts for each category and variable (second round).

Variable 1 Explain Consequences		Variable 2 Cause		Variable 3 Offers Advice		Variable 4 Recommendation	
Category	No. of Posts	Category	No. of Posts	Category	No. of Posts	Category	No. of Posts
1.1 Mentioned	24	2.1 Relationships	48	3.1 Directly offer advice	106	4.1 No recommendation	257
1.2 Not mentioned	399	2.2 Living sexuality	46	3.2 Advice is intuited	35	4.2 Self-compassion	31
		2.3 Access university	40	3.3 Advice based on studies	88	4.3 Therapy	32
		2.4 COVID-19	34	3.4 Not mentioned	274	4.4 Medicines	23
		2.5 Mental health	14			4.5 Meeting people	43
		2.6 Work	35			4.6 Change of job	8
		2.7 Masculinity	18			4.7 Other	61
		2.8 Childhood	16				
		2.9 Other	32				
		2.10 Not mentioned	143				

**Table 4:** Codification for messages specified in the results.

Post No.	Variable 1 Explain Consequences	Variable 2 Cause	Variable 3 Offers Advice	Variable 4 Recommendation
12	1.2 Not mentioned	2.10 Not mentioned	3.4 Not mentioned	4.4 Medicines
15	1.2 Not mentioned	2.2 Diverse ways of living sexuality	3.4 Not mentioned	4.1 No recommendation
43	1.2 Not mentioned	2.7 Masculinity	3.4 Not mentioned	4.1 No recommendation
45	1.2 Not mentioned	2.7 Masculinity	3.4 Not mentioned	4.1 No recommendation
46	1.2 Not mentioned	2.7 Masculinity	3.4 Not mentioned	4.1 No recommendation
47	1.2 Not mentioned	2.7 Masculinity	3.4 Not mentioned	4.1 No recommendation
48	1.2 Not mentioned	2.7 Masculinity	3.4 Not mentioned	4.1 No recommendation
49	1.2 Not mentioned	2.7 Masculinity	3.4 Not mentioned	4.1 No recommendation
66	1.2 Not mentioned	2.9 Other	3.1 Directly	4.2 Self-compassion
138	1.1 Mentioned	2.10 Not mentioned	3.4 Not mentioned	4.1 No recommendation
153	1.2 Not mentioned	2.7 Masculinity	3.4 Not mentioned	4.1 No recommendation
168	1.1 Not mentioned	2.10 Not mentioned	3.2 Intuited	4.5 Meeting people
169	1.1 Not mentioned	2.10 Not mentioned	3.4 Not mentioned	4.1 No recommendation
172	1.1 Not mentioned	2.2 Diverse ways of living sexuality	3.4 Not mentioned	4.1 No recommendation
179	1.1 Not mentioned	2.2 Diverse ways of living sexuality	3.4 Not mentioned	4.1 No recommendation
182	1.1 Not mentioned	2.2 Diverse ways of living sexuality	3.4 Not mentioned	4.1 No recommendation
184	1.2 Mentioned	2.2 Diverse ways of living sexuality	3.4 Not mentioned	4.1 No recommendation
223	1.1 Not mentioned	2.2 Diverse ways of living sexuality	3.4 Not mentioned	4.1 No recommendation
244	1.2 Mentioned	Not mentioned	3.4 Not mentioned	4.4. Medicines
245	1.1 Not mentioned	2.2 Diverse ways of living sexuality	3.4 Not mentioned	4.1 No recommendation
259	1.2 Mentioned	2.10 Not mentioned	3.4 Not mentioned	4.1 No recommendation
268	1.2 Mentioned	2.10 Not mentioned	3.4 Not mentioned	4.1 No recommendation
271	1.1 Not mentioned	2.9 Other	3.4 Not mentioned	4.1 No recommendation
274	1.1 Not mentioned	2.3 Access to university	3.4 Not mentioned	4.1 No recommendation
277	1.1 Not mentioned	2.3 Access to university	3.2 Intuited	4.2 Self-compassion
293	1.1 Not mentioned	2.9 Other	3.4 Not mentioned	4.1 No recommendation
301	1.2 Mentioned	2.10 Not mentioned	3.1 Directly	4.7 Other
411	1.2 Mentioned	2.10 Not mentioned	3.4 Not mentioned	4.1 No recommendation
459	1.1 Not mentioned	2.2 Diverse ways of living sexuality	3.4 Not mentioned	4.1 No recommendation
506	1.1 Not mentioned	2.10 Not mentioned	3.4 Not mentioned	4.1 No recommendation
537	1.1 Not mentioned	2.4 COVID-19	3.4 Not mentioned	4.1 No recommendation
545	1.1 Not mentioned	2.4 COVID-19	3.4 Not mentioned	4.1 No recommendation
605	1.1 Not mentioned	2.5 Diagnosis of mental health problems	3.4 Not mentioned	4.1 No recommendation
897	1.1 Not mentioned	2.10 Not mentioned	3.4 Not mentioned	4.1 No recommendation
910	1.1 Not mentioned	2.2 Diverse ways of living sexuality	3.4 Not mentioned	4.5 Meeting people
917	1.1 Not mentioned	2.10 Not mentioned	3.4 Not mentioned	4.1 No recommendation
939	1.1 Not mentioned	2.2 Diverse ways of living sexuality	3.4 Not mentioned	4.5 Meeting people
953	1.1 Not mentioned	2.2 Diverse ways of living sexuality	3.4 Not mentioned	4.3 Therapy
956	1.1 Not mentioned	2.10 Not mentioned	3.4 Not mentioned	4.5 Meeting people

## Data Availability

The data presented in this study are openly available in Zenodo at: https://doi.org/10.5281/zenodo.4729725 (accessed on 9 July 2021).
